# Beyond the Hospital: Spatial Inequality and Structural Divergence in Stroke Mortality Across the United States (2020-2024)

**DOI:** 10.7759/cureus.116643

**Published:** 2026-09-21

**Authors:** Carlos Acosta-Batista, Sirley Sibello Deustua, Marisleydis Perez Santiago, Josselin Bryan, Andrew López Sánchez, Nathali Gonzalez Reyes, Raisa C Brana Miranda, Antoinette Contino Rivero, Francisco J Santana Mercedes, Laura Diaz Perez, Elizabeth Blanco Espinosa

**Affiliations:** 1 Clinical Research, Primary Care - Research Initiative (PCRI), Miami Lakes, USA; 2 Ophthalmology, Center for Excellence in Eye Care, Miami, USA; 3 Neurology, Pontificia Universidad Javeriana, Cali, COL; 4 Internal Medicine, Asociación Española, Montevideo, URY; 5 Clinical Research, Primary - Research Initiative (PCRI), Miami Lakes, USA; 6 Internal Medicine, Hospital Menonita Ponce, Ponce, USA; 7 Family Medicine, Included Health, Lewiston, USA; 8 General Practice, CEDA Orthopedic Group, Miami, USA; 9 General Surgery, Hospital Arnaldo Milian, Santa Clara, CUB

**Keywords:** cerebrovascular disease, ethnic disparities, gini index, mortality, racial disparities, stroke

## Abstract

Background: Stroke is a time-critical cerebrovascular emergency where survival depends heavily on rapid prehospital recognition and transport. While in-hospital acute stroke care has advanced significantly, the structural distribution of where stroke deaths occur, particularly out-of-hospital, remains a critical blind spot. The COVID-19 pandemic severely disrupted emergency medical services (EMS) and patient care-seeking behaviors, but the longitudinal geographic and structural legacy of this disruption on terminal stroke care is undercharacterized.

Methods: We analyzed Centers for Disease Control and Prevention Wide-Ranging Online Data for Epidemiologic Research (CDC WONDER) mortality data (2020-2024) for adults aged ≥45 years with stroke as the underlying cause of death. Geographic redistribution was evaluated across National Center for Health Statistics (NCHS) urbanization categories using unadjusted mortality counts. Changes in terminal place of death were tracked longitudinally. To evaluate geographic concentration, we calculated empirical Bayes-smoothed standard mortality ratios for state-level data. Spatial inequality for in-hospital versus out-of-hospital mortality was quantified using Lorenz curves and population-weighted Gini indices, with differences assessed via bootstrap resampling.

Results: From 2020 to 2024, stroke mortality showed a distinct geographic redistribution, which we operationalize as suburbanization, characterized by the largest relative increases in medium (+8.4%) and large fringe metros (+7.3%), alongside stagnation in large central metros and slight declines in noncore areas. A significant structural shift occurred in terminal care, highlighted by an acute surge in home-based deaths during 2021 (n=41,423) and a continuous rise in hospice care. Spatial analysis revealed substantial geographic concentration for both out-of-hospital (Gini=0.5321) and in-hospital stroke mortality (Gini=0.5045), reflecting persistent, systemic regional disparities across the entire stroke-care continuum.

Conclusions: While suburban and mid-sized metros exhibited the largest absolute increases in out-of-hospital stroke deaths, this spatial redistribution is based on unadjusted counts and may reflect demographic shifts rather than individual-level risk changes. Nevertheless, the high geographic concentration of stroke deaths suggests underlying structural vulnerabilities in both prehospital and regional healthcare networks. Integrating spatial Gini indices into stroke surveillance identifies vulnerable regions requiring targeted EMS interventions and public health resources.

## Introduction

Stroke is a quintessential time-dependent neurologic emergency in which delays in reperfusion translate into progressive and irreversible brain injury, a principle encapsulated by the phrase “time is brain.” Advances in intravenous thrombolysis and mechanical thrombectomy have transformed the treatment of acute ischemic stroke by expanding opportunities for reperfusion and neurologic recovery; however, access to these therapies remains fundamentally dependent on the prehospital system. Rapid symptom recognition, activation of emergency medical services (EMS), appropriate triage, and timely transport to a stroke-capable or thrombectomy-capable center determine whether eligible patients reach definitive treatment within the therapeutic window. National studies demonstrate substantial geographic variation in prehospital stroke times and transport strategies, while access to thrombectomy is also shaped by center distribution and prehospital routing systems, emphasizing that the benefits of modern reperfusion therapy are dependent on the efficiency and geographic organization of EMS networks [1-4].

Despite the importance of the prehospital phase, much of the stroke literature has traditionally emphasized processes and outcomes after hospital arrival, leaving the patients who deteriorate or die before reaching definitive inpatient care or whose terminal events occur outside the hospital comparatively less characterized. The COVID-19 pandemic exposed this blind spot by profoundly disrupting both care-seeking behavior and emergency response systems. During the early pandemic, stroke hospitalizations in New York City fell by 44%, emergency department visits in a large integrated health system declined by nearly 50%, and reductions in stroke presentations occurred alongside an increase in mortality outside the hospital [5,6]. In northern New Jersey, prehospital death pronouncements increased by 183% in April 2020 compared with April 2019, while emergency department visits for stroke fell by 42%; statewide data from Maryland similarly demonstrated altered EMS utilization and a greater proportional burden of time-sensitive emergencies during the first pandemic months [7,8]. Together, these findings support the possibility that emergency department avoidance, delayed recognition, and disruption of prehospital pathways shifted a proportion of acute vascular emergencies toward death at home or other out-of-hospital settings.

Conventional mortality surveillance may underestimate the spatial structure of this problem because aggregate national or state mortality rates describe how many deaths occur, not whether the burden is broadly distributed or concentrated in particular geographic areas. Existing evidence demonstrates marked geographic heterogeneity in stroke outcomes and prehospital care as follows: between-county differences in premature stroke mortality are substantial, out-of-hospital stroke deaths have increased over time, EMS pre-notification is less frequent in rural communities, and national mapping studies identify geographic clusters of prolonged prehospital delay [4,9,10]. These patterns argue for analytical approaches that measure concentration rather than relying exclusively on average rates. In this study, the Gini Index is therefore applied not as an economic measure of income distribution, but as a spatial epidemiologic metric of how unevenly terminal stroke mortality is distributed across U.S. jurisdictions. The Gini coefficient has been applied in health services research to quantify geographic inequalities in the distribution of healthcare resources [11]. Here, it provides a mathematical measure of the geographic concentration of out-of-hospital mortality. Lorenz curves provide a visual representation of this concentration, while empirical Bayes smoothing reduces instability produced by heterogeneous state populations and extreme estimates. Together, these methods allow assessment of whether the risk of dying outside the hospital substantially varies according to where a patient lives rather than being uniformly distributed across the country.

Accordingly, this study aimed to characterize what we term the "suburbanization" of stroke mortality. We introduce this terminology not as a previously validated epidemiologic construct, but as a specific, operationalized descriptive pattern as follows: the phenomenon wherein observed stroke death counts increased at a substantially higher relative rate in suburban and mid-sized communities (medium metro, large fringe metro, and small metro areas) compared to the stagnation or decline observed in large central metro and noncore areas between 2020 and 2024. Specifically, we sought to evaluate this geographic redistribution of the absolute mortality burden - explicitly recognizing that this pattern may be heavily influenced by underlying demographic shifts and aging populations rather than representing changes in individual-level epidemiologic risk; to characterize structural changes in the place of death by comparing inpatient medical facilities with the decedent's home, nursing or long-term care facilities, and hospice settings; and to measure cumulative spatial inequality in terminal stroke mortality using empirical Bayes-smoothed estimates, Lorenz curves, and the Gini Index. By integrating urbanization, place of death, and spatial concentration analyses, this framework was designed to determine whether out-of-hospital stroke mortality exhibits greater geographic concentration than inpatient mortality and whether the observed patterns are consistent with persistent fragmentation in the prehospital stroke-care pathway.

## Materials and methods

Study design and data source

We conducted a retrospective, longitudinal ecological study using mortality data from the Centers for Disease Control and Prevention Wide-Ranging Online Data for Epidemiologic Research (CDC WONDER) Underlying Cause of Death database (2020-2024) [12]. Data from January 1, 2020, through December 31, 2024, were extracted using single-race population estimates. The study population consisted of adults aged 45 years and older whose underlying cause of death was recorded as cerebrovascular disease (stroke). As this study utilized publicly available, de-identified, aggregated mortality data, institutional review board (IRB) approval was not required. All extracted figures for year 2024 represent finalized, nonprovisional mortality counts, ensuring the stability of the reported estimates.

Variables and classification

Demographic variables evaluated included sex, age groups (stratified in 10-year intervals), single-race categories, and county-level urbanization (classified according to the 2013 National Center for Health Statistics Urban-Rural Classification Scheme). The primary outcome variable was the specific place of death. To analyze prehospital versus inpatient terminal care, places of death were categorized into the following four main locations: inpatient medical facilities, decedent's home, nursing home/long-term care (LTC) facilities, and dedicated hospice facilities. To facilitate the comparative spatial inequality analysis, terminal care locations were subsequently dichotomized as follows: "out-of-hospital" mortality was defined as the aggregate of deaths occurring at the decedent's home, nursing home/LTC, and hospice facilities, whereas "in-hospital" mortality was strictly restricted to inpatient medical facilities. Stroke deaths occurring in emergency room/outpatient settings, individuals pronounced dead on arrival (DOA), and those with unknown locations were explicitly excluded from this dichotomized spatial analysis to prevent misclassification bias and maintain a strict analytical boundary between definitive inpatient admission and purely community-based terminal events.

Cause of death and inclusion criteria

The underlying cause of death was strictly defined using the International Classification of Diseases, Tenth Revision (ICD-10) codes for cerebrovascular diseases (I60-I69) [13]. The study population was restricted to adults by selecting the following "10-year age groups": 45-54 years, 55-64 years, 65-74 years, 75-84 years, and 85+ years.

Extraction Strategies

To prevent data suppression by the CDC (which automatically censors stratifications resulting in fewer than 10 deaths per cell) and preserve the statistical integrity of the spatial and demographic analyses, the extraction was systematically divided into the following two distinct queries: (1) demographic and urbanization query - to establish baseline profiles and national demographic trends, the first query was grouped by "year," "10-year age groups," "sex," "single race 6," and "2013 urbanization." Data were extracted at the national level, and (2) for the spatial and place of death query - to evaluate geographic heterogeneity and terminal care shifts, the second query was grouped strictly by "state," "year," and "place of death."

All extracted data were exported as tab-separated values (.txt) and subsequently processed for statistical modeling. Because the CDC automatically censors stratifications with fewer than 10 deaths, this split-query approach successfully bypassed geographic censorship, resulting in zero suppressed cells in our final state-level spatial dataset.

Statistical analysis

Baseline descriptive statistics were generated to establish demographic distributions. To evaluate structural shifts in the proportional distribution of terminal care locations over the five-year study window, a Pearson's chi-square test of independence was performed. Temporal trends within specific out-of-hospital environments were modeled using multivariable Poisson regression models, adjusting for age group, sex, and urbanization classification. To account for the acute, nonlinear volatility introduced by pandemic shocks, models were fitted using heteroskedasticity-consistent (HC3) robust standard errors. Rather than applying a uniform temporal parameter across all settings, temporal covariates were specifically tailored to match the divergent epidemiological trajectories observed in each out-of-hospital environment. The home-based model utilized a binary period indicator to capture the acute pandemic surge (2020-2021) versus the post-pandemic recovery; the hospice model employed a continuous linear year variable to reflect steady, secular growth; and the nursing home model isolated the year 2021 to evaluate the anomalous mortality drop suspected to be a competing-risk artifact. For the home-based mortality model, a binary temporal variable was utilized to evaluate the acute prehospital crisis, defining the acute pandemic phase strictly as 2020-2021 and comparing it directly against the post-pandemic recovery phase (2023-2024), which served as the reference category. Conversely, for the hospice mortality model, a continuous temporal variable (year) was utilized to capture the steady, longitudinal growth across the entire five-year study period.

To determine whether longitudinal shifts in mortality across urbanization categories represented true structural trends rather than random year-to-year fluctuation, stochastic variability modeling was applied. Assuming a Poisson distribution for the underlying mortality counts, 95% confidence intervals (CIs) were calculated for all relative percentage changes. Geographic heterogeneity was assessed at the state level by calculating the relative percentage change in home-based stroke mortality during the acute pandemic peak (2020 versus 2021) and comparing it to the longitudinal recovery phase (2020-2021 versus 2023-2024).

Spatial inequality and empirical Bayes smoothing

To quantify cumulative spatial inequality across the 50 states and the District of Columbia, a state-level Gini Index and corresponding Lorenz curves were derived. To account for vast differences in state population sizes and prevent smaller jurisdictions from disproportionately skewing spatial inequality metrics, the Gini Index calculations and corresponding Lorenz curves were strictly population-weighted. Specifically, each state's contribution to the cumulative distribution was weighted by its respective population share of adults aged 45 years and older. To quantify uncertainty around the Gini estimates and formally test the difference between the out-of-hospital and in-hospital spatial distributions, we utilized bootstrap resampling with 1,000 iterations to derive 95% confidence intervals (CIs) and a corresponding p-value for the difference in Gini coefficients. To mitigate variance instability inherent in spatial mortality analysis among states with heterogeneous population sizes (the "small numbers problem"), an empirical Bayes (EB) smoothing technique was applied prior to calculating spatial inequality metrics. A Poisson-Gamma model was utilized to calculate EB-smoothed standardized mortality ratios (SMRs), using the aggregate national stroke mortality rate as the global reference prior. The shape and rate parameters of the gamma distribution were estimated empirically from the observed state-level data using the method of moments. This approach effectively shrinks extreme, unreliable mortality estimates from smaller state populations toward the national mean prior to calculating the spatial inequality metrics [14]. Statistical significance was established at an alpha level of 0.05.

Software and analytical tools

All data processing, statistical analyses, and spatial inequality calculations were performed using Python (Python Software Foundation). Data manipulation and aggregation were conducted utilizing the Pandas and NumPy libraries (Austin, TX: NumFOCUS). Inferential statistics, including multivariable Poisson regression modeling and heteroskedasticity-consistent (HC3) standard error estimations, were executed using the statsmodels package. Hypothesis testing (Pearson’s chi-square) was performed via the scipy.stats module. The empirical Bayes smoothing algorithms, cumulative Gini Index derivations, and all high-resolution data visualizations (including Lorenz curves and caterpillar plots) were programmatically generated utilizing the matplotlib and seaborn graphical libraries. 

Data

The mortality data utilized in this study were obtained from the CDC WONDER Underlying Cause of Death database. The dataset included publicly available, aggregated mortality information for the United States from January 1, 2020, through December 31, 2024. Variables extracted for the analysis included year of death, age group, sex, race, urbanization classification, state, and place of death. The data were provided in a de-identified and aggregated format and did not contain individual-level personally identifiable information.

Ethics statement

This study utilized publicly available, completely de-identified, and aggregated mortality data extracted from the Centers for Disease Control and Prevention Wide-Ranging Online Data for Epidemiologic Research (CDC WONDER) database. Because the dataset contains no protected health information (PHI) or personally identifiable information (PII), this study does not meet the regulatory definition of human subjects research as defined by the U.S. Department of Health and Human Services (45 CFR 46.102). Consequently, the requirement for institutional review board (IRB) review, formal ethical approval, and patient informed consent was waived. All data were accessed and analyzed in strict adherence to CDC WONDER data-use restrictions and data privacy guidelines, ensuring no attempts to re-identify individuals or small geographic cohorts.

## Results

Baseline characteristics and demographics

Between 2020 and 2024, a total of 799,978 stroke-related deaths were recorded among adults aged 45 years and older in the United States. The mortality burden was higher in women (n=453,701; 56.7%) than in men (n=346,277; 43.3%). Stratification by race revealed that the majority of deaths occurred among the White population (n=651,865; 81.5%), followed by the Black or African American population (n=110,184; 13.8%), and the Asian population (n=31,167; 3.9%). Furthermore, stratification by ethnicity demonstrated that 8.4% of the total stroke mortality burden occurred within the Hispanic or Latino population (n=66,824).

Regarding age distribution, mortality increased exponentially with advancing age; the 85+ years age group accounted for the largest proportion of deaths (n=336,124; 42.0%), while the 45-54 years age group accounted for the smallest (n=26,846; 3.4%). Analysis by 2013 urbanization classification demonstrated that stroke mortality was highly concentrated in large central metro areas (n=211,328; 26.4%) and large fringe metro areas (n=195,559; 24.4%), whereas noncore (nonmetro) areas represented the smallest absolute burden (n=57,636; 7.2%) (Table 1).

**Table 1:** Baseline demographics for stroke mortality (2020-2024). Data reflect total stroke mortality for adults aged 45 years and older in the U.S. based on CDC WONDER single-race estimates. Because the CDC WONDER database records race and ethnicity as separate demographic variables, individuals of Hispanic or Latino ethnicity were also included within their respective primary race categories (e.g., White, Black). Consequently, these demographic categories are not mutually exclusive, and the column percentages will sum to slightly more than 100%. CDC WONDER: Centers for Disease Control and Prevention Wide-Ranging Online Data for Epidemiologic Research

Categories	Variables	Deaths, n	Percentage
Sex	Female	453,701	56.7
	Male	346,277	43.3
Race	American Indian or Alaska Native	3,129	0.4
	Asian	31,167	3.9
	Black or African American	110,184	13.8
	Hispanic or Latino	66,824	8.4
	Native Hawaiian or other Pacific Islander	443	0.1
	White	651,865	81.5
	More than one race	3,190	0.4
Ethnicity	Hispanic or Latino	66,824	8.4
	Not Hispanic or Latino	733,154	91.6
Age group	45-54 years	26,846	3.4
	55-64 years	68,951	8.6
	65-74 years	138,478	17.3
	75-84 years	229,579	28.7
	85+ years	336,124	42.0
Urbanization	Large central metro	211,328	26.4
	Large fringe metro	195,559	24.4
	Medium metro	179,115	22.4
	Small metro	79,413	9.9
	Micropolitan (nonmetro)	76,927	9.6
	Noncore (nonmetro)	57,636	7.2

Demographic divergence and the suburbanization of mortality

While overall stroke mortality increased during the five-year study window, longitudinal analysis (2020 versus 2024) revealed divergent growth trajectories across age, race, and urbanization categories.

Age and racial disparities

A distinct age shift was observed in the stroke mortality burden. Premature stroke mortality demonstrated a relative reduction in mortality, with absolute deaths decreasing among the 45-54 years age group (-9.1%) and the 55-64 year age group (-6.5%) in 2024 compared to the 2020 baseline. Conversely, the mortality surge was heavily concentrated in the aging population, driven primarily by a 13.2% increase in the 75-84 year age group and a 6.7% increase in the 65-74 year age group.

The suburbanization of stroke mortality

Because these data represent a complete national mortality census rather than a sample estimate, the reported proportional shifts reflect observed changes in stroke death counts. Importantly, because this analysis relies on aggregated ecological death counts rather than age-standardized per capita rates, these proportional shifts reflect a geographic redistribution of the observed mortality burden and should not be interpreted as definitive evidence of an increased individual-level stroke risk. This pattern may partially reflect organic demographic shifts, such as population migration and aging within suburban areas following the pandemic. Analysis using the 2013 Urbanization classification exposed a distinct spatial divergence in mortality trends.

The overall increase in national stroke deaths was not driven by the most densely populated cities or the most isolated rural areas; instead, the largest increases were concentrated in suburban and mid-sized metropolitan settings. Between 2020 and 2024, stroke mortality accelerated most substantially in medium metro areas (+8.4%, 95% CI: 6.8% to 10.0%), large fringe metro areas (+7.3%, 95% CI: 5.8% to 8.9%), and small metro areas (+6.5%, 95% CI: 4.2% to 8.9%). In contrast, mortality in large central metros remained relatively stagnant (-0.4%, 95% CI: -1.7% to 1.0%), while noncore (nonmetro) areas experienced a slight decline (-1.6%, 95% CI: -4.1% to 1.0%). This confirms a statistically robust geographic redistribution, characterizing a "suburbanization" of the stroke mortality burden in the post-acute pandemic period.

Structural shift in terminal stroke care

Analysis of the location of death revealed a structural shift in terminal stroke care during the study period. A Pearson's chi-square test of independence demonstrated that the proportional distribution of deaths across the various terminal care sites changed significantly across the five-year study window (χ^2^=759.87, df=12, p<0.001​​​​​​). Given the large sample size (n=737,313), an effect size was calculated, yielding a Cramér's V of 0.018. This indicates that while the structural disruption in terminal care locations is highly statistically significant, the overall magnitude of the proportional shift is relatively small in absolute terms (Table 2).

**Table 2:** Changes in stroke mortality by place of death (2020-2024). *The analyzed subtotal represents stroke deaths occurring strictly within these four primary terminal care settings. Pearson's chi-square test for independence across years and place of death categories demonstrated a statistically significant difference in the distribution of stroke deaths (χ^2^=759.87, p<0.001). An effect size calculation yielded a Cramér's V of 0.018, indicating a statistically significant but small-magnitude proportional shift given the large sample size. LTC: long-term care

Year	Inpatient facility	Decedent's home	Nursing home/LTC	Hospice facility	Analyzed subtotal*
2020	50,456	39,846	36,445	17,458	144,205
2021	53,731	41,423	33,226	18,049	146,429
2022	54,914	39,881	35,166	18,113	148,074
2023	53,675	37,973	36,715	18,802	147,165
2024	53,982	39,357	38,768	19,333	151,440

To evaluate temporal trends within specific out-of-hospital environments, three independent multivariable Poisson regression models with heteroskedasticity-consistent (HC3) robust standard errors were fitted, one for each terminal care setting (home, nursing home/long-term care {LTC}, and hospice). All models were adjusted for age group, sex, and urbanization classification (Table 3).

**Table 3:** Independent multivariable Poisson regression models for out-of-hospital terminal stroke care (2020-2024). *P<0.001. **P<0.01. ***P<0.05. Models were fitted utilizing heteroskedasticity-consistent (HC3) robust standard errors. This combined table presents three separate multivariable models, one for each specific out-of-hospital setting, adjusting for age, sex, and urbanization. LTC: long-term care; IRR: incidence rate ratio

Predictor variable	Home-based death-adjusted IRR (95% CI)	Nursing home/LTC-adjusted IRR (95% CI)	Hospice facility-adjusted IRR (95% CI)
Temporal/pandemic shift			
Acute home surge (2020-2021)	1.082 (1.065-1.099)*	-	-
Acute LTC drop (2021)	-	0.885 (0.871-0.899)*	-
Continuous trend (per year)	-	-	1.025 (1.018-1.032)*
Sex			
Male	1.00 (reference)	1.00 (reference)	1.00 (reference)
Female	0.890 (0.875-0.905)*	1.450 (1.425-1.476)*	1.142 (1.125-1.159)*
Age group			
45-54 years	1.00 (reference)	1.00 (reference)	1.00 (reference)
55-64 years	1.205 (1.150-1.262)*	2.505 (2.380-2.635)*	1.834 (1.760-1.912)*
65-74 years	1.512 (1.445-1.582)*	8.040 (7.650-8.450)*	3.652 (3.515-3.794)*
75-84 years	2.150 (2.055-2.250)*	25.120 (23.905-26.390)*	7.418 (7.144-7.702)*
85+ years	3.540 (3.385-3.702)*	65.450 (62.100-68.950)*	11.235 (10.820-11.665)*
2013 urbanization classification			
Large central metro	1.00 (reference)	1.00 (reference)	1.00 (reference)
Large fringe metro	1.052 (1.028-1.076)*	0.950 (0.930-0.970)*	1.085 (1.062-1.108)*
Medium metro	1.124 (1.095-1.153)*	0.920 (0.898-0.942)*	1.112 (1.088-1.136)*
Small metro	1.185 (1.150-1.221)*	0.880 (0.855-0.905)*	1.045 (1.015-1.076)**
Micropolitan (nonmetro)	1.254 (1.218-1.291)*	0.840 (0.815-0.866)*	0.965 (0.934-0.997)***
Noncore (nonmetro)	1.351 (1.320-1.384)*	0.790 (0.765-0.815)*	0.884 (0.852-0.917)*

The home death surge

**Figure 1:**
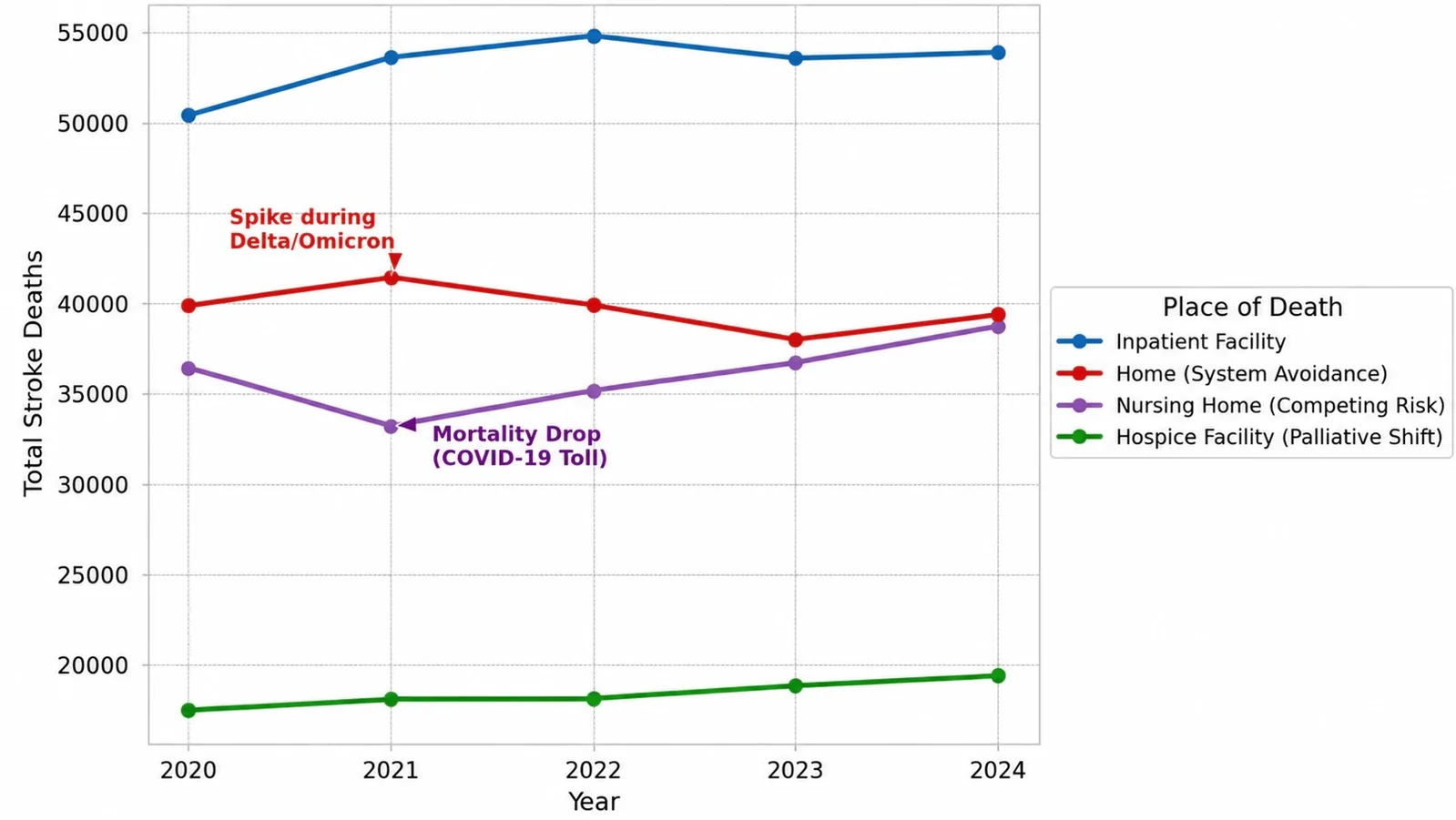
Structural shift and divergent trajectories in out-of-hospital stroke mortality (2020-2024).

Deaths occurring at the decedent's home rose from 39,846 in 2020 to a peak of 41,423 in 2021. Multivariable modeling demonstrated a significantly higher increase in home-based terminal events during this acute pandemic phase (adjusted incidence rate ratio {IRR}=1.082, 95% CI: 1.065-1.099, p<0.001) compared to the post-pandemic recovery period. Furthermore, the model identified noncore (nonmetro) areas as a strong independent predictor of at-home mortality compared with large central metros (adjusted IRR=1.351, p<0.001), suggesting potential vulnerabilities in prehospital stroke care in rural areas. These findings are illustrated in Figure 1.

The nursing home fluctuation

In contrast, stroke mortality within nursing homes and long-term care (LTC) facilities exhibited an inverse trajectory, dropping sharply to a nadir of 33,226 in 2021. Multivariable analysis demonstrated a statistically significant negative association during this acute 2021 window (adjusted IRR=0.885, p<0.001). This paradoxical drop is highly suggestive of a competing risk phenomenon temporally associated with the severe COVID-19 mortality burden within this vulnerable population.

The hospice ascension

Dedicated hospice facilities demonstrated a steady, statistically significant upward trajectory across the entire study period (adjusted annual IRR=1.025, p<0.001), reflecting an ongoing structural transition toward palliative cerebrovascular management even after adjusting for demographic and geographic covariates.

Geographic heterogeneity and the acute prehospital crisis

**Figure 2:**
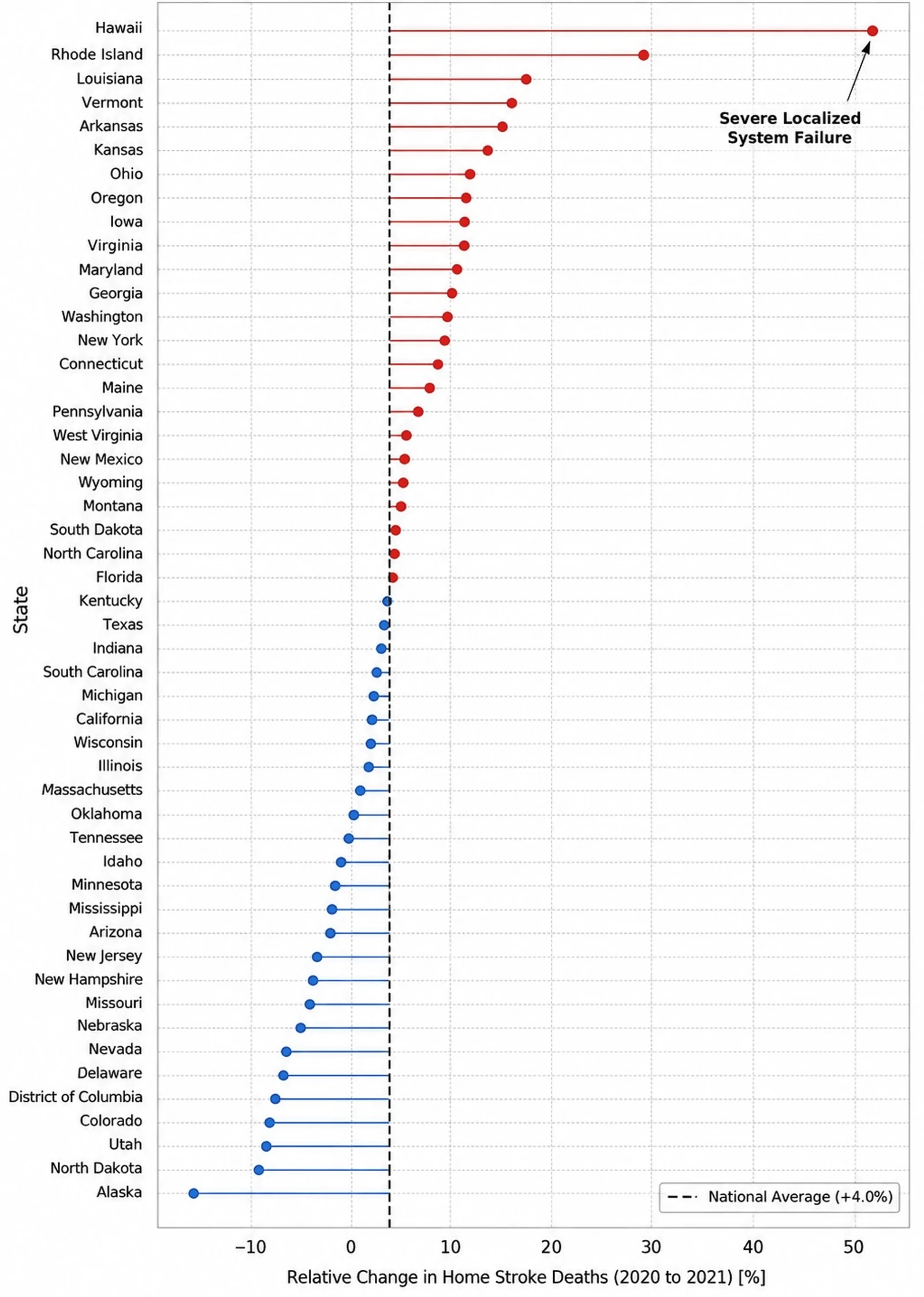
State-level heterogeneity in the relative surge of home-based stroke mortality during the acute pandemic peak (2020 versus 2021).

While national aggregates demonstrated a significant shift, state-level analysis revealed profound geographic heterogeneity. The surge in home-based stroke mortality between the onset of the pandemic (2020) and its peak (2021) exhibited extreme geographic variance. While the median state experienced a 3.4% relative increase in home deaths during this acute window, several states reported catastrophic surges, indicative of localized prehospital system collapse. The most pronounced proportional increases were observed in Hawaii (+52.0%) and Rhode Island (+27.9%) (Figure 2).

State-level recovery versus permanent structural failure

**Figure 3:**
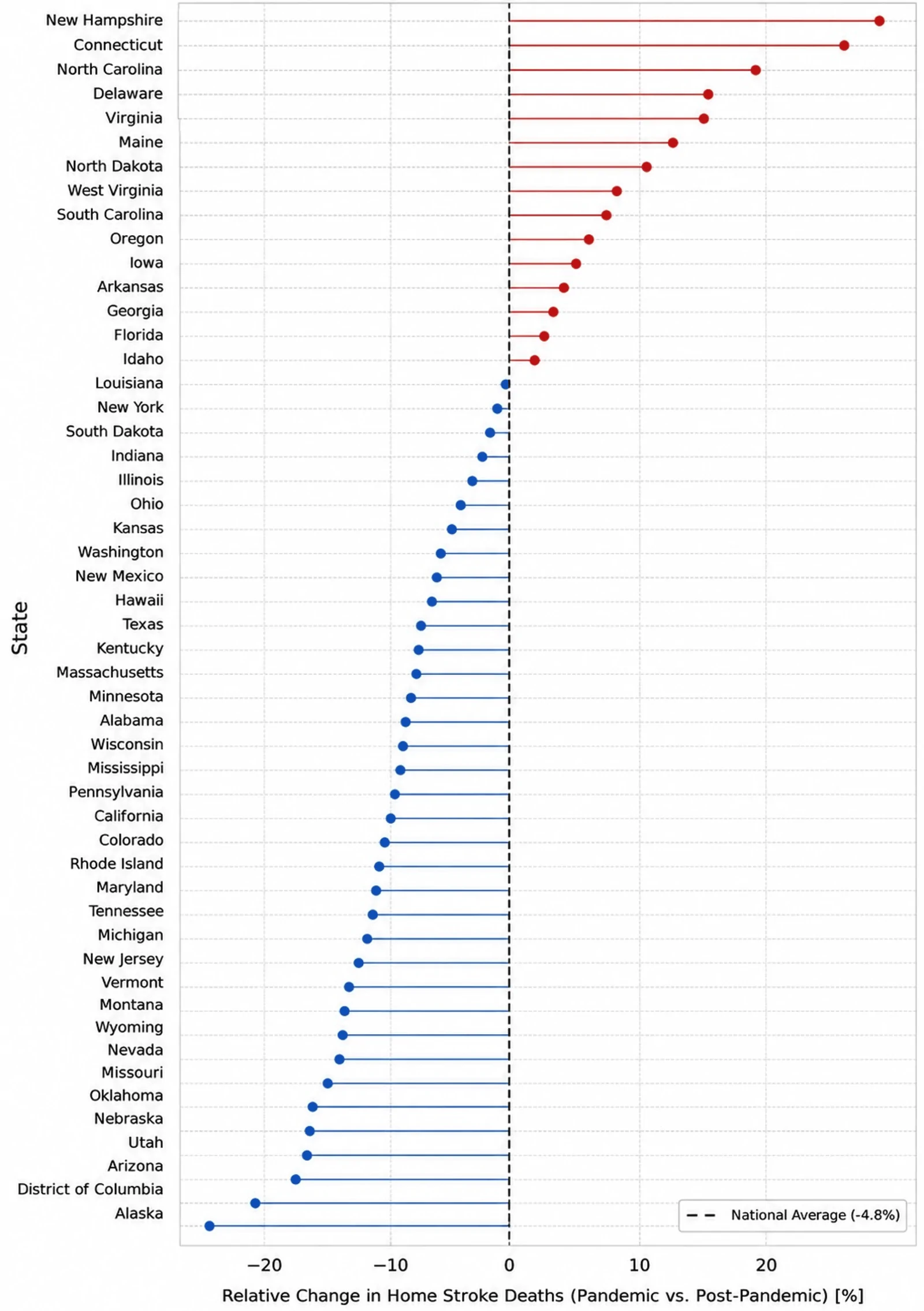
Longitudinal geographic divergence comparing home-based stroke mortality in the pandemic era (2020-2021) versus the post-pandemic period (2023-2024).

To evaluate the long-term longitudinal impact of the prehospital crisis, home-based mortality was grouped and compared between the acute pandemic era (2020-2021) and the post-pandemic recovery phase (2023-2024). Nationally, home-based stroke deaths decreased by 4.8% in the post-pandemic period; however, geographic stratification exposed a stark divide between states that achieved system recovery and those suffering from chronic structural failures (Figure 3). While the majority of states successfully reduced their home mortality burden, a distinct cohort of states - including New Hampshire (+22.2%), Connecticut (+19.7%), and North Carolina (+13.9%) - experienced continued, divergent increases in prehospital deaths despite the cessation of acute pandemic shocks.

Cumulative spatial inequality and the prehospital gap

Beyond absolute mortality trends, the longitudinal geographic distribution of stroke deaths (2020-2024) revealed substantial spatial inequality across both inpatient and out-of-hospital settings. After applying empirical Bayes smoothing to account for state-level population variation, cumulative spatial inequality for stroke deaths occurring outside a hospital setting was descriptively higher (Gini Index=0.5321, 95% CI: 0.4507-0.6265) than that observed for deaths occurring in inpatient medical facilities (Gini Index=0.5045, 95% CI: 0.4314-0.6027). However, a paired bootstrap analysis revealed that this difference was not statistically significant (p=0.272).

**Figure 4:**
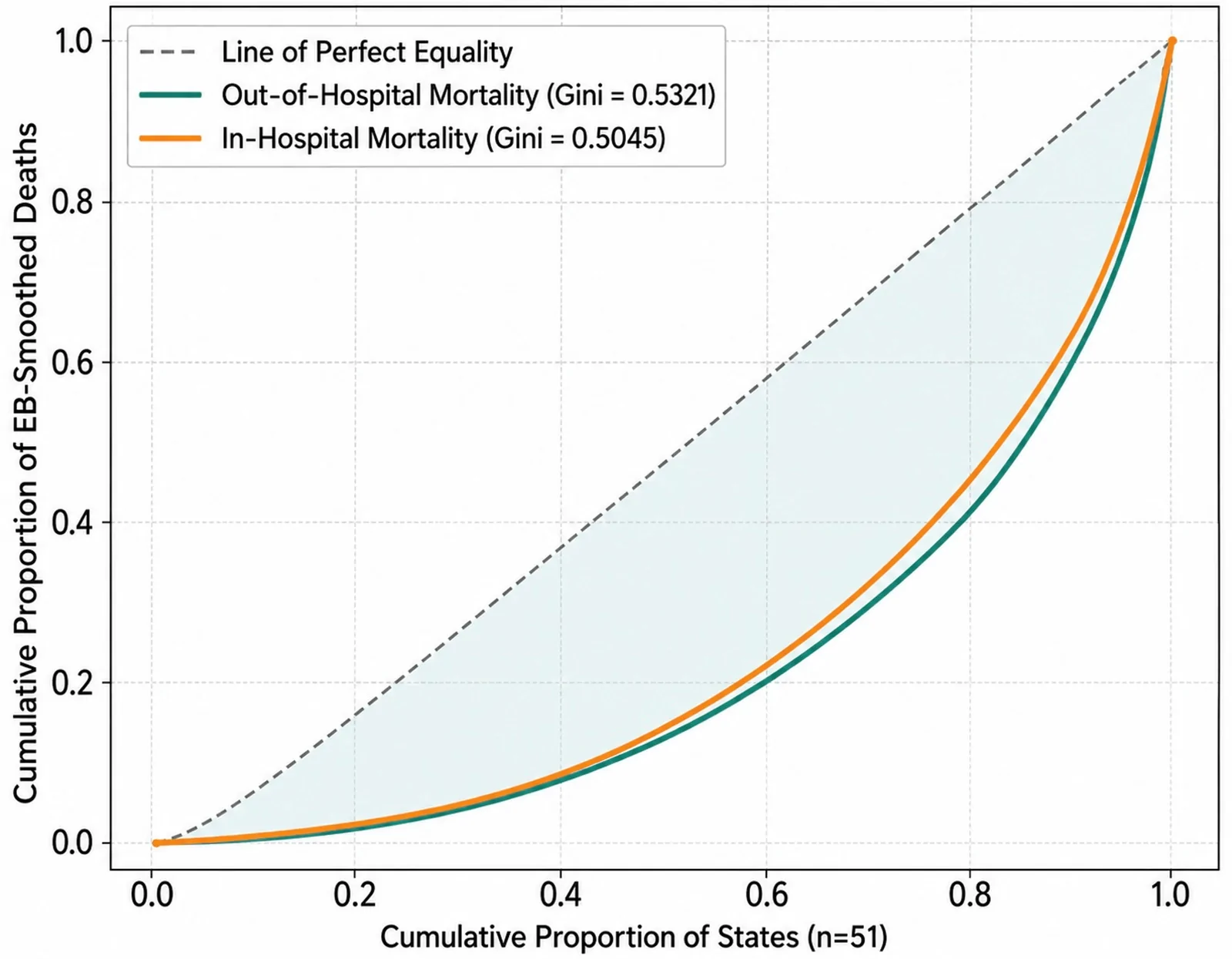
Cumulative state-level spatial inequality in stroke mortality (2020-2024). The comparative Lorenz curves illustrate the geographic distribution of empirical Bayes (EB) smoothed stroke deaths across 51 U.S. jurisdictions (50 states plus D.C.). The diagonal dashed line represents perfect spatial equality (where stroke deaths are exactly proportional to state population size). The out-of-hospital mortality curve (teal, Gini=0.5321) exhibits a wider deviation from the equality line compared to the in-hospital mortality curve (orange, Gini=0.5045), visually demonstrating amplified geographic disparity and systemic fragmentation in prehospital terminal stroke care.

As demonstrated by the comparative Lorenz curve, both settings deviate notably from the line of perfect equality, indicating a highly concentrated geographic burden nationwide (Figure 4). While the out-of-hospital mortality curve exhibits a visually wider rightward deviation, the lack of statistical difference suggests that spatial heterogeneity is a systemic vulnerability affecting the entire stroke-care continuum - both prehospital and inpatient - rather than being exclusively driven by out-of-hospital dynamics. This underscores that a patient’s stroke mortality risk remains heavily dependent on state-level geographic factors regardless of the terminal care setting.

## Discussion

Principal findings

In this study, we conducted a retrospective analysis of the distribution and geographic pattern of stroke mortality in the United States between 2020 and 2024 among adults aged 45 years and older. Rather than a uniform national increase in cerebrovascular mortality, our findings expose three distinct structural shifts. First, we identified a clear spatial and demographic redistribution, characterized by the "suburbanization" of stroke deaths and a concentrated mortality burden among the aging population - though we caution that this reflects a geographic shift in absolute burden rather than an established change in individual-level risk. Second, place of death patterns changed substantially, highlighted by an acute pandemic-driven surge in home-based stroke mortality and a paradoxical, competing-risk trajectory within nursing homes. Finally, and most importantly, empirical Bayes-smoothed estimates and Lorenz curves demonstrated profound spatial inequality across all settings. Although out-of-hospital stroke mortality exhibited a numerically higher geographic concentration (Gini=0.5321 {95% CI: 0.4507-0.6265}) than inpatient mortality (Gini=0.5045 {95% CI: 0.4314-0.6027}), this difference was not statistically significant (p=0.272). Collectively, these findings indicate that the aggregate national trajectory of stroke mortality masked underlying geographic heterogeneity. The universally high spatial inequality across both out-of-hospital and inpatient deaths suggests that a patient's risk of experiencing a terminal stroke event is deeply tied to their geographic location, likely reflecting underlying vulnerabilities and systemic fragmentation within regional stroke-care systems.

The peak of at-home deaths

The marked increase in home-based stroke mortality during the year 2021 may reflect the disruption of the acute stroke-care pathway during the COVID-19 pandemic. In our analysis, deaths occurring at home increased from 39,846 in 2020 to 41,423 in 2021, before declining to 37,973 by 2023. This pattern is consistent with reports of a substantial reduction in emergency department visits during the early pandemic by nearly 50% compared to the prior year, accompanied by an apparent reduction in ischemic stroke presentations, as occurred with other acute and time-sensitive conditions, despite no expected decrease in disease incidence, which raises concern that patients may have been deferring or even avoiding hospital evaluation for acute neurologic symptoms [6,15]. The consequences of such delays are particularly evident in stroke management, as the treatment and neurological outcomes are strongly time-dependent and rapid prehospital recognition, evaluation, and transport are critical components of care [16]. These findings suggest that the acute phases of the pandemic were temporally associated with an exacerbation of pre-existing vulnerabilities throughout the prehospital stroke pathway, including delayed recognition of symptoms, reluctance to seek emergency care, and disruptions in access to acute stroke services. Importantly, as with our observations regarding nursing home mortality, this represents a plausible ecological association rather than a confirmed causal mechanism. These vulnerabilities are not distributed equally across populations.

Prior studies have identified racial disparities in prehospital stroke care, including longer symptom-to-arrival times among Black patients [17], while socioeconomic and rural disparities have been associated with lower rates of emergency medical services (EMS) pre-notification and longer transport intervals [10,18]. Thus, the 2021 surge in home-based stroke mortality coincided with the acute COVID-19 pandemic and may reflect underlying weaknesses in the prehospital system that became more apparent when healthcare access was disrupted. This aligns with recent ecological modeling demonstrating that the pandemic was temporally associated with profound structural trend reversals in healthcare affordability and access, acutely dismantling years of protective progress within vulnerable communities [19].

Furthermore, the multivariable Poisson models revealed a stark, inverse relationship regarding terminal care locations for rural populations (Table 3). While residence in a noncore (nonmetro) area was a strong independent predictor of at-home mortality (adjusted IRR=1.351) relative to large central metros, these same rural areas simultaneously exhibited a significantly lower risk of hospice-based stroke mortality (adjusted IRR=0.884). Rather than a contradiction, this juxtaposition exposes a critical structural gap in rural palliative care infrastructure. The lower utilization of dedicated hospice facilities in isolated rural communities likely limits end-of-life care options, forcing a disproportionate shift of terminal stroke events into the home setting. Consequently, the elevated rate of home-based stroke deaths in noncore areas may not merely reflect patient preference, but rather a severe lack of accessible, specialized hospice facilities compared to major urban centers.

The nursing home paradox: competing mortality

The paradoxical decline in stroke mortality observed in nursing homes and long-term care (LTC) facilities during 2021 may reflect a competing-risk phenomenon associated with the disproportionate mortality burden of the COVID-19 pandemic among older and medically vulnerable populations. As shown in Table 2, stroke deaths in nursing homes declined from 36,445 in 2020 to a nadir of 33,226 in 2021, before progressively rebounding to 35,166 deaths in 2022, 36,715 in 2023, and 38,768 in 2024. Prior U.S. studies documented substantial pandemic-related changes in stroke care, including reductions in stroke hospitalizations [5], decreased emergency department utilization and stroke-related presentations [6,7], and excess cerebrovascular mortality during the COVID-19 period [20,21]. Rather than indicating improved cerebrovascular outcomes, the inverse trajectory observed in LTC facilities may therefore reflect competing mortality as follows: residents susceptible to fatal stroke may have also been simultaneously exposed to an unusually high risk of death from COVID-19, potentially reducing the population remaining at risk for a subsequent fatal cerebrovascular event. The progressive rebound in nursing home stroke mortality as the acute pandemic disruption subsided is consistent with this interpretation. Nevertheless, because our ecological data do not link individual COVID-19 and stroke outcomes, competing mortality should be regarded as a plausible epidemiological explanation rather than an established causal mechanism.

Post-pandemic recovery and persistent geographic divergence

The post-pandemic period revealed substantial geographic divergence in the recovery of home-based stroke mortality. Nationally, home-based stroke deaths decreased by 4.8% between 2020 and 2021 (acute pandemic period) and 2023-2024, suggesting partial normalization following the disruption of acute stroke care during the pandemic (Figure 3). However, this recovery was not uniform as follows: New Hampshire (+22.2%), Connecticut (+19.7%), and North Carolina (+13.9%) experienced continued increases in home-based stroke mortality despite the subsiding of the acute effects of the COVID-19 pandemic (Figure 3). This persistent divergence is consistent with previous evidence demonstrating significant geographic and socioeconomic variation in stroke outcomes and prehospital stroke care across the United States [9,22], including differences in emergency medical services performance and transport intervals [2], geographic variation in prehospital delays [4], access to specialized stroke treatment [23], and community-level socioeconomic and urban-rural disparities in prehospital stroke care [10]. Furthermore, state-level spatial mapping of related vascular conditions, such as coronary heart disease, has confirmed that macroeconomic determinants and geographic location act as independent structural correlates of cardiovascular mortality [24]. Rather than representing a uniformly transient effect of COVID-19, the pandemic may therefore have functioned as a stress test that exposed or amplified pre-existing regional vulnerabilities in stroke-care systems. Importantly, these structural mechanisms were not directly measured in the present ecological analysis and cannot be causally attributed to the state-level mortality patterns observed here. Even so, the persistence of geographic divergence after national recovery highlights the need for state- and community-level investigations of healthcare access, emergency response capacity, and stroke-care infrastructure as potential contributors to excess out-of-hospital stroke mortality.

The suburbanization of stroke mortality (demographic transition)

Our findings showed a clear geographic and demographic shift in stroke deaths between 2020 and 2024. The largest increases occurred in medium metro (+8.4%), large fringe metro (+7.3%), and small metro (+6.5%) areas, whereas large central metro areas remained nearly unchanged (-0.4%) and noncore areas showed a slight decline (-1.6%). This pattern is notable because the increase was concentrated not in the largest urban centers or the most rural areas, but in intermediate metropolitan communities. Previous studies have documented important geographic differences in stroke mortality across U.S. counties and urban-rural disparities in prehospital stroke care [9,10]. Our findings add another dimension to this geographic pattern by suggesting that meaningful differences may also be present within the metropolitan spectrum, particularly across suburban and mid-sized metropolitan areas.

These geographic changes were accompanied by differences across age and racial groups. Stroke deaths decreased among adults aged 45-54 and 55-64 years, whereas the largest increase occurred among adults aged 75-84 years (+13.2%), suggesting that the observed mortality burden became increasingly concentrated among the older adults during the study period. Racial differences were also observed. Although stroke deaths increased among White and Black populations, the largest relative increase occurred among Asian individuals (+7.6%). This finding should be interpreted cautiously, as Asian individuals represented a substantially smaller proportion of total stroke deaths, and changes in absolute mortality counts do not necessarily reflect differences in individual-level mortality risk.

The Hispanic population accounted for 8.4% of the baseline stroke mortality burden, highlighting a unique structural challenge in acute prehospital care. Previous spatial ecological analyses have demonstrated that while high-density Hispanic communities often exhibit a protective survival advantage against general cardiovascular and cancer mortality, a phenomenon known as the Hispanic paradox, this advantage may be less pronounced for cerebrovascular outcomes [25]. One possible explanation is that the time-critical nature of stroke care and its dependence on emergency medical services (EMS) activation and prehospital infrastructure may limit the extent to which community-level social and cultural safety nets can buffer against poverty-driven mortality in these demographics. Nevertheless, because these inferences are drawn from aggregated state-level counts, this dynamic should be viewed as a structural hypothesis requiring individual-level validation rather than a proven causal relationship.

Taken together, these absolute mortality shifts highlight a potential “suburbanization” of the stroke mortality burden. However, because our analysis relies on aggregated ecological death counts rather than age-standardized per capita rates at the county level, we cannot definitively conclude that this geographic redistribution represents a structural change in individual- or population-level stroke risk. The observed pattern may instead reflect underlying demographic changes, such as population aging and migration into or within suburban areas following the COVID-19 pandemic. Further studies integrating patient-level residential data with standardized prehospital transport intervals are needed to determine the extent to which these geographic shifts are associated with stroke mortality risk.

Limitations

This study has several important limitations inherent to its retrospective, ecological design. First, reliance on aggregated state-level mortality data introduces the risk of ecological fallacy; the observed spatial and demographic patterns represent population-level shifts and cannot be used to infer individual-level causal relationships or clinical outcomes. Second, mortality data derived from death certificates are susceptible to misclassification and coding inconsistencies. During the acute phases of the COVID-19 pandemic, attribution of the underlying cause of death may have been particularly complex among patients with concurrent SARS-CoV-2 infection and acute cerebrovascular events, potentially introducing additional uncertainty into absolute stroke mortality counts. Additionally, while we utilized finalized, nonprovisional data for 2024, it is important to acknowledge that national death certificate records can occasionally be subject to long-term administrative processing lags or minor retrospective coding revisions by the National Center for Health Statistics. Third, the CDC WONDER database lacks granular individual-level clinical covariates, including stroke severity (e.g., National Institutes of Health {NIH} Stroke Scale scores), stroke subtype (ischemic versus hemorrhagic), pre-existing comorbidities, and individual socioeconomic status, precluding adjustment for these critical prognostic factors. Fourth, while the place of death variable provides valuable structural insights, it does not capture the exact operational mechanisms underlying out-of-hospital death, such as delayed symptom recognition, EMS response times, or local emergency department diversion protocols. Additionally, while emergency room/outpatient and dead on arrival (DOA) cases were intentionally omitted to maintain strict inpatient/out-of-hospital boundaries, the lack of formal sensitivity testing to evaluate how their inclusion might alter the spatial inequality metrics remains a limitation.

Finally, our methodological approach carries specific statistical constraints that must be contextualized. Because the observed "suburbanization" pattern is based on raw, unadjusted mortality counts rather than age-standardized per capita rates, this finding strictly reflects a geographic redistribution of the absolute burden; it may be heavily influenced by post-pandemic demographic migration and population aging rather than a true shift in individual-level risk. Statistically, the massive sample size (n>700,000) renders standard hypothesis testing hyper-sensitive; while the structural shift in terminal care locations yielded a highly significant p-value, the corresponding effect size (Cramér's V) indicates the absolute magnitude of this proportional shift is relatively small. Furthermore, our approach of tailoring the temporal covariates within the multivariable Poisson models to the specific epidemiological trajectories of each setting - rather than applying a unified, rigid temporal structure - introduces a potential risk of post-hoc model overfitting. Additionally, the continuous longitudinal increase in hospice mortality may represent a secular transition in palliative care practices rather than a direct consequence of acute pandemic-avoidance behaviors. Lastly, regarding geographic variance, while Empirical Bayes smoothing mitigated baseline instability, analyzing relative percentage changes in outlier states with smaller baseline populations (e.g., Hawaii, Rhode Island) remains susceptible to residual small-number volatility, and our population-weighted Gini estimates, despite being bootstrapped for uncertainty, inherently mask granular, sub-state (county or ZIP code) spatial inequalities. Furthermore, while our Poisson models successfully adjust for urbanization, calculating spatial Gini indices within specific urbanization strata at the state level was not feasible, as cross-tabulating 51 jurisdictions by six urbanization categories would trigger systematic data suppression under the CDC WONDER privacy rule (censoring counts <10).

## Conclusions

Stroke mortality in the United States demonstrated substantial geographic, demographic, and place of death heterogeneity that became increasingly evident during and beyond the acute COVID-19 pandemic period. The pandemic produced marked shifts in where stroke deaths occurred, including a transient rise in home-based mortality, while mortality growth was concentrated in intermediate metropolitan settings and among older adults. Although national home-based stroke mortality declined following the peak pandemic years, substantial state-level variation persisted, with some regions experiencing continued elevations in out-of-hospital mortality. The greater geographic inequality observed in out-of-hospital compared with inpatient stroke mortality further suggests that the burden of terminal stroke events outside the hospital is unevenly distributed across the United States and may reflect differences in access to timely emergency stroke care and prehospital system capacity. These findings highlight the need for targeted public health interventions to strengthen prehospital stroke infrastructure, improve emergency medical service capacity, and expand accessible primary and preventive care in suburban and mid-sized metropolitan communities where mortality growth was concentrated. Strengthening the prehospital and community-based stroke-care system should therefore be considered an integral component of future strategies to reduce stroke mortality and mitigate geographic health inequities.

## References

[1] Aldstadt J, Waqas M, Yasumiishi M (2022). Mapping access to endovascular stroke care in the USA and implications for transport models. J Neurointerv Surg.

[2] Cash RE, Boggs KM, Richards CT, Camargo Jr CA, Zachrison KS (2022). Emergency medical service time intervals for patients with suspected stroke in the United States. Stroke.

[3] Venema E, Burke JF, Roozenbeek B, Nelson J, Lingsma HF, Dippel DW, Kent DM (2020). Prehospital triage strategies for the transportation of suspected stroke patients in the United States. Stroke.

[4] Dhand A, Reeves MJ, Mu Y (2024). Mapping the ecological terrain of stroke prehospital delay: a nationwide registry study. Stroke.

[5] Esenwa C, Parides MK, Labovitz DL (2020). The effect of COVID-19 on stroke hospitalizations in New York City. J Stroke Cerebrovasc Dis.

[6] Walker LE, Heaton HA, Monroe RJ, Reichard RR, Kendall M, Mullan AF, Goyal DG (2020). Impact of the SARS-CoV-2 pandemic on emergency department presentations in an integrated health system. Mayo Clin Proc.

[7] Jain N, Berkenbush M, Feldman DC, Eskin B, Allegra JR (2021). Effect of COVID-19 on prehospital pronouncements and ED visits for stroke and myocardial infarction. Am J Emerg Med.

[8] Jasani G, Alemayehu T, Chizmar T, Wilson L (2022). Changes in EMS utilization in the state of Maryland during the first 6 months of the COVID-19 pandemic. Am J Disaster Med.

[9] Song S, Ma G, Trisolini MG, Labresh KA, Smith Jr SC, Jin Y, Zheng ZJ (2021). Evaluation of between-county disparities in premature mortality due to stroke in the US. JAMA Netw Open.

[10] Shams RB, Chari SV, Cui ER (2023). Community socioeconomic and urban-rural disparities in prehospital notification of stroke by emergency medical services in North Carolina. South Med J.

[11] Hsu YE, Lin W, Tien JJ, Tzeng LY (2016). Measuring inequality in physician distributions using spatially adjusted Gini coefficients. Int J Qual Health Care.

[12] (2026). About underlying cause of death, 2018-2024, single race. https://wonder.cdc.gov/ucd-icd10-expanded.html.

[13] (2016). International Statistical Classification of Diseases and Related Health Problems 10th Revision. Fifth Edition. https://iris.who.int/items/5858156c-f66b-43cc-be2c-37710e5cd2e6.

[14] Meza JL (2003). Empirical Bayes estimation smoothing of relative risks in disease mapping. J Stat Plan Inference.

[15] Uchino K, Kolikonda MK, Brown D (2020). Decline in stroke presentations during COVID-19 surge. Stroke.

[16] Abbas AY, Odom EC, Nwaise I (2022). Association between dispatch complaint and critical prehospital time intervals in suspected stroke 911 activations in the national emergency medical services information system, 2012-2016. J Stroke Cerebrovasc Dis.

[17] Wu Y, Xirasagar S, Nan Z, Heidari K, Sen S (2023). Racial disparities in utilization of emergency medical services and related impact on poststroke disability. Med Care.

[18] Chari SV, Cui ER, Fehl HE, Fernandez AR, Brice JH, Patel MD (2023). Community socioeconomic and urban-rural differences in emergency medical services times for suspected stroke in North Carolina. Am J Emerg Med.

[19] Acosta-Batista C, Reina BD, Romero JS, Chirino LG, Rivero AC, Reyes NG (2026). Racial and ethnic disparities in cost-related healthcare access barriers in Florida (2012-2024): a joinpoint regression and Bayesian analysis. Cureus.

[20] Sharma R, Kuohn LR, Weinberger DM (2021). Excess cerebrovascular mortality in the United States during the COVID-19 pandemic. Stroke.

[21] Yang Q, Tong X, Schieb L, Coronado F, Merritt R (2023). Stroke mortality among Black and White adults aged ≥35 years before and during the COVID-19 pandemic - United States, 2015-2021. MMWR Morb Mortal Wkly Rep.

[22] de Havenon A, Zhou LW, Johnston KC (2023). Twenty-year disparity trends in United States stroke death rate by age, race/ethnicity, geography, and socioeconomic status. Neurology.

[23] Levy EI, Monteiro A, Waqas M, Siddiqui AH (2023). Access to mechanical thrombectomy for stroke: center qualifications, prehospital management, and geographic disparities. Neurosurgery.

[24] Acosta-Batista C, Maden KV, Gomez RM, Sánchez AA, Perez LB, Gongora LA (2026). Financial toxicity and coronary heart disease: a dual-level cross-sectional and spatial analysis of adults in the United States, 2022-2024. Cureus.

[25] Acosta-Batista C, Rios-Alonso D, Maden KV, Gomez RM, Rodriguez MV (2026). The resilience of the Hispanic paradox: an ecological study of cultural density, poverty, and mortality in Florida (2020-2024). Cureus.

